# 3-Benzyl-7-bromo-9-phenyl-2-tosyl-2,3,3a,4,9,9a-hexa­hydro-1*H*-pyrrolo[3,4-*b*]quinoline

**DOI:** 10.1107/S1600536809045875

**Published:** 2009-11-07

**Authors:** K. Chinnakali, D. Sudha, M. Jayagobi, R. Raghunathan, Hoong-Kun Fun

**Affiliations:** a Department of Physics, Anna University Chennai, Chennai 600 025, India; b Department of Organic Chemistry, University of Madras, Guindy Campus, Chennai 600 025, India; c X-ray Crystallography Unit, School of Physics, Universiti Sains Malaysia, 11800 USM, Penang, Malaysia

## Abstract

In the title compound, C_31_H_29_BrN_2_O_2_S, the pyrrolidine ring is in a twist conformation and the tetra­hydro­pyridine ring adopts an envelope conformation with the methine C atom adjacent to the NH group as the flap atom; the two rings are *trans*-fused. The bromo­benzene ring and the nearest phenyl ring form a dihedral angle of 82.72 (10)°. The benzyl phenyl and the tosyl phenyl rings are oriented at a dihedral angle of 75.57 (11)°. An intra­molecular N—H⋯π inter­action is observed. In the crystal, mol­ecules are linked into chains running along [101] by C—H⋯O hydrogen bonds and the chains are cross-linked *via* weak C—H⋯π inter­actions.

## Related literature

For the biological activity of pyrroloquinoline derivatives, see: Peng *et al.* (2002 ▶); Metobo *et al.* (2009 ▶); Ferlin *et al.* (2005 ▶); Ryu *et al.* (2009 ▶); Tsuji *et al.* (1995 ▶); Ferlin *et al.* (2001 ▶). For the crystal structures of chlorine and unbrominated analogues, see: Chinnakali *et al.* (2009*a* ▶,*b* ▶). For ring puckering parameters, see: Cremer & Pople (1975 ▶). For asymmetry parameters, see: Duax *et al.* (1976 ▶).
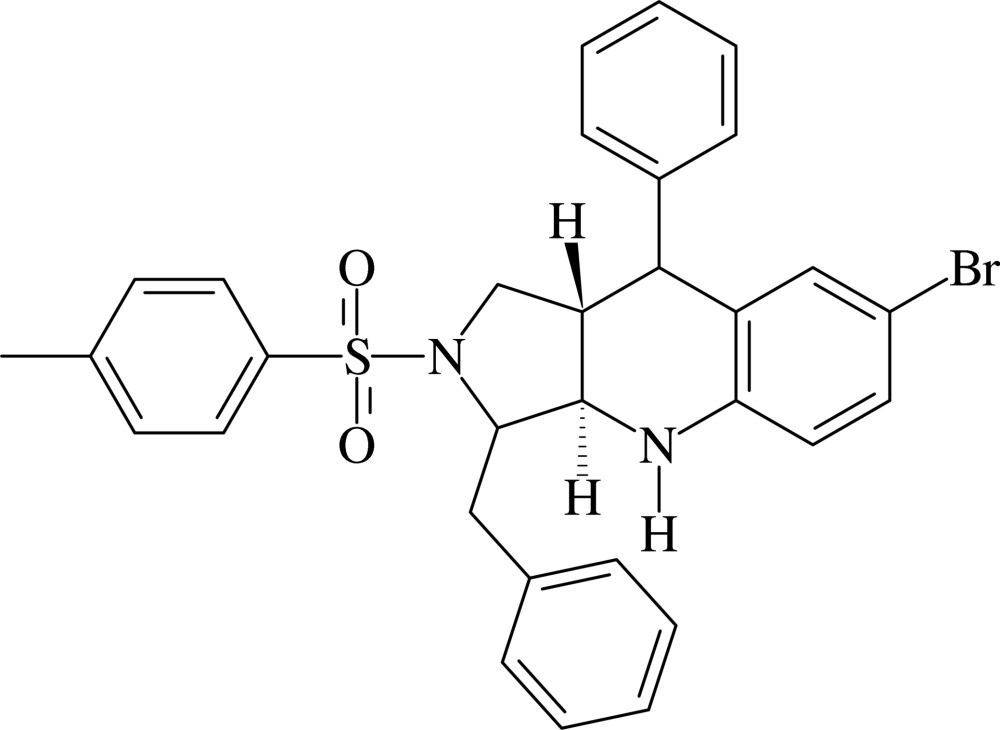


## Experimental

### 

#### Crystal data

C_31_H_29_BrN_2_O_2_S
*M* *_r_* = 573.53
Monoclinic, 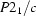

*a* = 8.8992 (2) Å
*b* = 27.5824 (5) Å
*c* = 13.3668 (2) Å
β = 127.190 (1)°
*V* = 2613.78 (9) Å^3^
*Z* = 4
Mo *K*α radiation
μ = 1.69 mm^−1^
*T* = 100 K
0.39 × 0.28 × 0.13 mm

#### Data collection

Bruker SMART APEXII CCD diffractometer
Absorption correction: multi-scan (*SADABS*; Bruker, 2005 ▶) *T*_min_ = 0.641, *T*_max_ = 0.805
43436 measured reflections
7624 independent reflections
6348 reflections with *I* > 2σ(*I*)
*R*_int_ = 0.034

#### Refinement

*R*[*F* ^2^ > 2σ(*F* ^2^)] = 0.040
*wR*(*F* ^2^) = 0.096
*S* = 1.06
7624 reflections
339 parameters
H atoms treated by a mixture of independent and constrained refinement
Δρ_max_ = 0.82 e Å^−3^
Δρ_min_ = −0.71 e Å^−3^

### 

Data collection: *APEX2* (Bruker, 2005 ▶); cell refinement: *SAINT* (Bruker, 2005 ▶); data reduction: *SAINT*; program(s) used to solve structure: *SHELXTL* (Sheldrick, 2008 ▶); program(s) used to refine structure: *SHELXTL*; molecular graphics: *SHELXTL*; software used to prepare material for publication: *SHELXTL* and *PLATON* (Spek, 2009 ▶).

## Supplementary Material

Crystal structure: contains datablocks global, I. DOI: 10.1107/S1600536809045875/hb5202sup1.cif

Structure factors: contains datablocks I. DOI: 10.1107/S1600536809045875/hb5202Isup2.hkl

Additional supplementary materials:  crystallographic information; 3D view; checkCIF report

## Figures and Tables

**Table 1:** Hydrogen-bond geometry (Å, °)

*D*—H⋯*A*	*D*—H	H⋯*A*	*D*⋯*A*	*D*—H⋯*A*
C28—H28⋯O2^i^	0.93	2.57	3.207 (3)	126
N2—H1*N*2⋯*Cg*3	0.83 (3)	2.53 (3)	3.289 (2)	152 (3)
C3—H3⋯*Cg*3^ii^	0.98	2.98	3.924 (2)	162
C18—H18*A*⋯*Cg*2^iii^	0.96	2.94	3.737 (3)	141
C21—H21⋯*Cg*1^iv^	0.93	2.80	3.676 (2)	158

Symmetry codes: (i) 
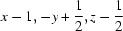
; (ii) 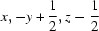; (iii) 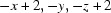; (iv) 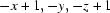. *Cg*1, *Cg*2 and *Cg*3 are the centroids of the C4–C9, C12–C17 and C26–C31 rings, respectively.

**Fig. 1.**
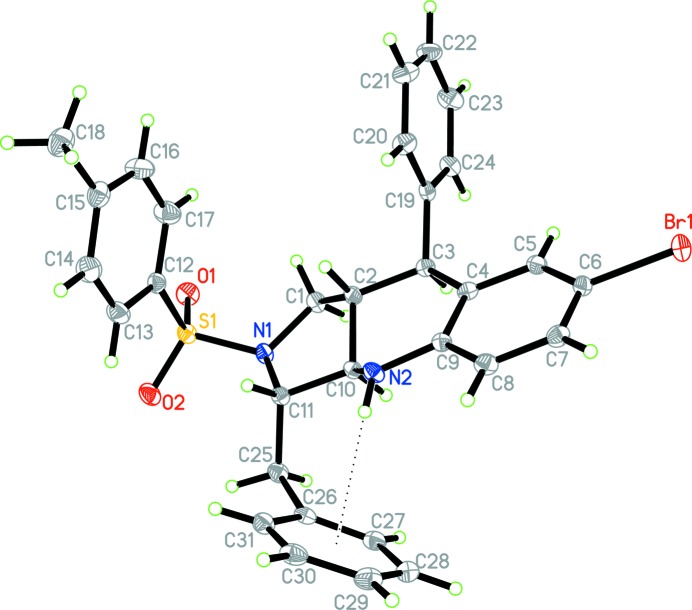
The molecular structure of (I), showing 50% probability displacement ellipsoids. The dotted line indicates an N—H···π interaction.

**Fig. 2.**
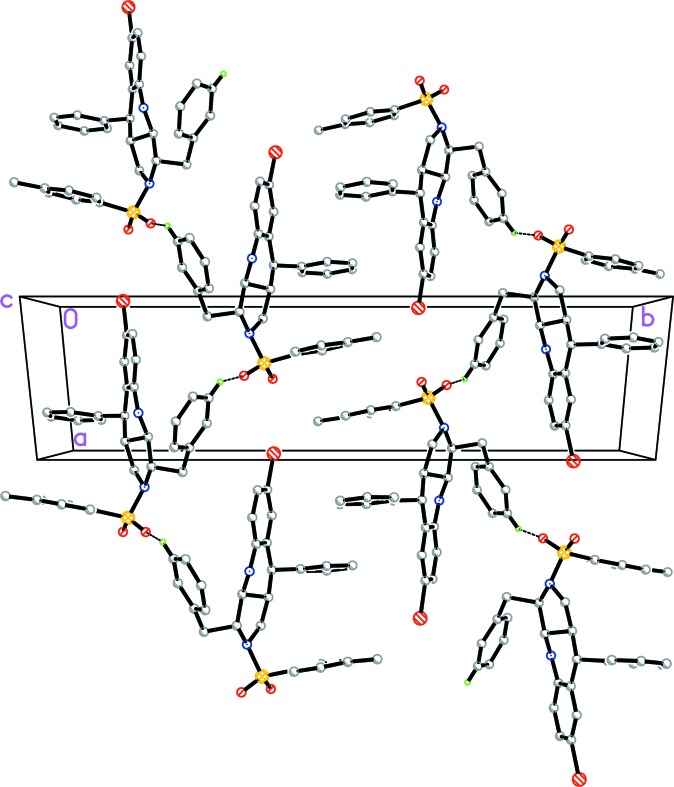
The crystal structure of (I), viewed along the c axis, shows chains running along the [101]. Hydrogen bonds are shown as dashed lines. For the sake of clarity, H atoms not involved in the interactions have been omitted.

**Fig. 3.**
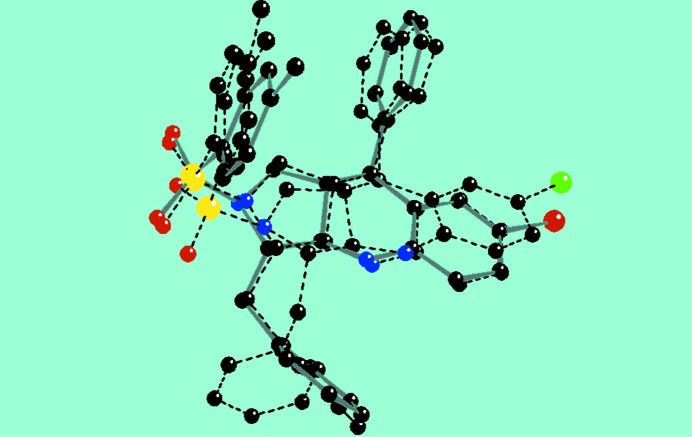
Super-position fit of (I) (solid lines), with chlorinated (Cl in green) and unchlorinated analogues (dashed lines). H atoms have been omitted for clarity.

## supplementary crystallographic information

### Comment

Pyrroloquinoline derivatives act as potent inhibitors for PI3-kinase related kinases (Peng *et al.*, 2002) and as HIV integrase inhibitors (Metobo *et al.*, 2009). These derivatives have been investigated as potential anticancer drugs (Ferlin *et al.*, 2005) and are found to exhibit antifungal (Ryu *et al.*, 2009), antibacterial (Tsuji *et al.*, 1995) and antiproliferative (Ferlin *et al.*, 2001) activities. As part of our studies on pyrroloquinoline derivatives, we report here the crystal structure of the title compound.

Bond lengths and angles are comparable with those in chlorine (Chinnakali *et al.*, 2009*a*) and unbrominated (Chinnakali *et al.*, 2009*b*) analouges. The pyrrolidine ring adopts a twist conformation, with puckering parameters (Cremer & Pople, 1975) q_2_ = 0.408 (2) Å and φ = 82.2 (3)°. The tetrahydropyridine ring adopts an envelope conformation with C10, the envelope flap, lying 0.713 (2) Å out of the plane formed by the rest of the atoms (N2/C2—C4/C9) of the ring (r.m.s. deviation 0.053 Å). The asymmetry parameter (Duax *et al.*, 1976) ΔC_s_[C10] = 12.1 (2)° and the puckering parameters (Cremer & Pople, 1975) Q = 0.537 (2) Å, θ = 128.5 (2)° and φ = 103.2 (3)°. The dihedral angle between the C4—C9 and C19—C24 rings is 82.72 (10)° and that between the C12—C17 and C26—C31 rings is 75.57 (11)°.

The molecules are linked into chains running along the [101] by C—H···O hydrogen bonds (Fig.2). The chains are cross-linked into a three-dimensional network *via* C—H···π interactions (Table 2) involving the C4—C9, C12—C17 and C26—C31 rings.

A superposition of the non-H atoms of the chlorine (Chinnakali *et al.*, 2009*a*) and unbrominated (Chinnakali *et al.*, 2009*b*) analouges with those of the title molecule using *XP* in *SHELXTL* (Sheldrick, 2008) is shown in Fig.3. The title molecule fits well (r.m.s. deviation 0.415 Å) with the unbrominated analouge. But the chlorine and bromine analouges differ significantly in the orientations of the benzyl group. In the title molecule as well as in the unbrominated derivative, the benzyl phenyl rings is oriented in such a way to form an N—H···π interaction. But in the chlorine analouge, the benzyl group is twisted away from the N—H group to form an N—H···Cl hydrogen bond.

### Experimental

InCl_3_ (20 mol%) was added to a mixture of *S*-2-(*N*-cinnamyl-*N*-tosylamino)-3-phenyl propanal (1 mmol) and *p*-bromoaniline (1 mmol) in acetonitrile (20 ml). The reaction mixture was stirred at room temperature for 1 min. On completion of the reaction, as indicated by TLC, the mixture was quenched with water and extracted with ethyl acetate. The organic layer was washed with brine and dried over Na_2_SO_4_. The solvent was evaporated *in vacuo* and the crude product was chromatographed on silica gel using a hexane-ethyl acetate (8.5:1.5 *v*/*v*) mixture to obtain the title compound. Colourless blocks of (I) were recrystallized from ethyl acetate solution by slow evaporation.

### Refinement

The N-bound H atom was located in a difference map and refined freely [N—H = 0.83 (3) Å]. The remaining H atoms were positioned geometrically (C—H = 0.93–0.98 Å) and allowed to ride on their parent atoms, with *U*_iso_(H) = 1.2*U*_eq_(C) or 1.5*U*_eq_(C_methyl_). A rotating group model was used for methyl groups.

### Crystal data

**Table:**

C_31_H_29_BrN_2_O_2_S	*F*(000) = 1184
*M**_r_* = 573.53	*D*_x_ = 1.457 Mg m^−^^3^
Monoclinic, *P*2_1_/*c*	Mo *K*α radiation, λ = 0.71073 Å
Hall symbol: -P 2ybc	Cell parameters from 9860 reflections
*a* = 8.8992 (2) Å	θ = 2.4–37.0°
*b* = 27.5824 (5) Å	µ = 1.69 mm^−^^1^
*c* = 13.3668 (2) Å	*T* = 100 K
β = 127.190 (1)°	Block, colourless
*V* = 2613.78 (9) Å^3^	0.39 × 0.28 × 0.13 mm
*Z* = 4	

### Data collection

**Table:**

Bruker SMART APEXII CCD diffractometer	7624 independent reflections
Radiation source: fine-focus sealed tube	6348 reflections with *I* > 2σ(*I*)
graphite	*R*_int_ = 0.034
φ and ω scans	θ_max_ = 30.0°, θ_min_ = 2.1°
Absorption correction: multi-scan (*SADABS*; Bruker, 2005)	*h* = −11→12
*T*_min_ = 0.641, *T*_max_ = 0.805	*k* = −35→38
43436 measured reflections	*l* = −18→16

### Refinement

**Table:**

Refinement on *F*^2^	Primary atom site location: structure-invariant direct methods
Least-squares matrix: full	Secondary atom site location: difference Fourier map
*R*[*F*^2^ > 2σ(*F*^2^)] = 0.040	Hydrogen site location: inferred from neighbouring sites
*wR*(*F*^2^) = 0.096	H atoms treated by a mixture of independent and constrained refinement
*S* = 1.06	*w* = 1/[σ^2^(*F*_o_^2^) + (0.0447*P*)^2^ + 2.281*P*] where *P* = (*F*_o_^2^ + 2*F*_c_^2^)/3
7624 reflections	(Δ/σ)_max_ = 0.001
339 parameters	Δρ_max_ = 0.82 e Å^−^^3^
0 restraints	Δρ_min_ = −0.71 e Å^−^^3^

### Special details

**Table:**

Geometry. All e.s.d.'s (except the e.s.d. in the dihedral angle between two l.s. planes) are estimated using the full covariance matrix. The cell e.s.d.'s are taken into account individually in the estimation of e.s.d.'s in distances, angles and torsion angles; correlations between e.s.d.'s in cell parameters are only used when they are defined by crystal symmetry. An approximate (isotropic) treatment of cell e.s.d.'s is used for estimating e.s.d.'s involving l.s. planes.
Refinement. Refinement of *F*^2^ against ALL reflections. The weighted *R*-factor *wR* and goodness of fit *S* are based on *F*^2^, conventional *R*-factors *R* are based on *F*, with *F* set to zero for negative *F*^2^. The threshold expression of *F*^2^ > σ(*F*^2^) is used only for calculating *R*-factors(gt) *etc*. and is not relevant to the choice of reflections for refinement. *R*-factors based on *F*^2^ are statistically about twice as large as those based on *F*, and *R*- factors based on ALL data will be even larger.

### Fractional atomic coordinates and isotropic or equivalent isotropic displacement parameters (Å^2^)

**Table:**

	*x*	*y*	*z*	*U*_iso_*/*U*_eq_	
Br1	−0.02354 (3)	0.121549 (7)	0.223962 (19)	0.01926 (6)	
S1	1.38753 (6)	0.137930 (17)	0.96077 (4)	0.01528 (10)	
O1	1.4895 (2)	0.12512 (5)	0.91282 (14)	0.0208 (3)	
O2	1.46953 (19)	0.17022 (5)	1.06590 (13)	0.0203 (3)	
N1	1.1910 (2)	0.16359 (6)	0.84519 (15)	0.0142 (3)	
N2	0.7082 (2)	0.15745 (6)	0.74366 (15)	0.0142 (3)	
H1N2	0.698 (4)	0.1741 (10)	0.791 (3)	0.029 (7)*	
C1	1.0890 (3)	0.14242 (7)	0.71672 (18)	0.0162 (4)	
H1A	1.1538	0.1140	0.7175	0.019*	
H1B	1.0766	0.1659	0.6582	0.019*	
C2	0.8972 (3)	0.12908 (6)	0.68243 (17)	0.0132 (3)	
H2	0.9089	0.0988	0.7250	0.016*	
C3	0.7325 (3)	0.12458 (6)	0.54317 (17)	0.0131 (3)	
H3	0.7436	0.1510	0.4990	0.016*	
C4	0.5476 (3)	0.13198 (6)	0.52621 (17)	0.0128 (3)	
C5	0.3766 (3)	0.12455 (6)	0.40768 (18)	0.0147 (3)	
H5	0.3776	0.1149	0.3414	0.018*	
C6	0.2054 (3)	0.13134 (6)	0.38721 (18)	0.0148 (4)	
C7	0.2001 (3)	0.14504 (7)	0.48506 (19)	0.0167 (4)	
H7	0.0853	0.1487	0.4717	0.020*	
C8	0.3676 (3)	0.15308 (7)	0.60239 (19)	0.0160 (4)	
H8	0.3647	0.1623	0.6681	0.019*	
C9	0.5425 (3)	0.14763 (6)	0.62462 (17)	0.0129 (3)	
C10	0.8645 (2)	0.17057 (7)	0.74190 (17)	0.0125 (3)	
H10	0.8312	0.1998	0.6908	0.015*	
C11	1.0543 (2)	0.17848 (7)	0.87013 (17)	0.0133 (3)	
H11	1.0652	0.1564	0.9316	0.016*	
C12	1.3304 (3)	0.08391 (7)	1.00181 (18)	0.0159 (4)	
C13	1.3157 (3)	0.08395 (8)	1.09972 (19)	0.0217 (4)	
H13	1.3314	0.1126	1.1416	0.026*	
C14	1.2775 (3)	0.04104 (8)	1.1346 (2)	0.0247 (4)	
H14	1.2671	0.0413	1.1998	0.030*	
C15	1.2546 (3)	−0.00222 (8)	1.0741 (2)	0.0203 (4)	
C16	1.2670 (3)	−0.00143 (8)	0.9752 (2)	0.0255 (4)	
H16	1.2495	−0.0300	0.9326	0.031*	
C17	1.3049 (3)	0.04111 (8)	0.9388 (2)	0.0245 (4)	
H17	1.3132	0.0409	0.8727	0.029*	
C18	1.2250 (3)	−0.04916 (8)	1.1177 (2)	0.0256 (4)	
H18A	1.1229	−0.0455	1.1232	0.038*	
H18B	1.3381	−0.0575	1.1988	0.038*	
H18C	1.1952	−0.0744	1.0589	0.038*	
C19	0.7354 (2)	0.07718 (7)	0.48649 (17)	0.0132 (3)	
C20	0.7079 (3)	0.03291 (7)	0.52376 (19)	0.0192 (4)	
H20	0.6864	0.0324	0.5837	0.023*	
C21	0.7122 (3)	−0.01038 (7)	0.4725 (2)	0.0227 (4)	
H21	0.6936	−0.0396	0.4982	0.027*	
C22	0.7443 (3)	−0.00997 (8)	0.3832 (2)	0.0233 (4)	
H22	0.7474	−0.0389	0.3489	0.028*	
C23	0.7717 (3)	0.03359 (8)	0.3452 (2)	0.0234 (4)	
H23	0.7941	0.0339	0.2857	0.028*	
C24	0.7656 (3)	0.07699 (7)	0.39584 (18)	0.0170 (4)	
H24	0.7820	0.1062	0.3688	0.020*	
C25	1.0855 (3)	0.23088 (7)	0.91806 (18)	0.0163 (4)	
H25A	1.2055	0.2335	1.0008	0.020*	
H25B	1.0872	0.2527	0.8618	0.020*	
C26	0.9258 (3)	0.24439 (7)	0.92394 (18)	0.0149 (3)	
C27	0.7728 (3)	0.27179 (7)	0.82900 (18)	0.0171 (4)	
H27	0.7786	0.2870	0.7693	0.021*	
C28	0.6109 (3)	0.27661 (7)	0.82256 (19)	0.0190 (4)	
H28	0.5094	0.2948	0.7585	0.023*	
C29	0.6011 (3)	0.25435 (7)	0.9116 (2)	0.0209 (4)	
H29	0.4921	0.2568	0.9062	0.025*	
C30	0.7551 (3)	0.22835 (7)	1.00898 (19)	0.0196 (4)	
H30	0.7502	0.2139	1.0698	0.023*	
C31	0.9168 (3)	0.22381 (7)	1.01571 (19)	0.0182 (4)	
H31	1.0201	0.2069	1.0821	0.022*	

### Atomic displacement parameters (Å^2^)

**Table:**

	*U*^11^	*U*^22^	*U*^33^	*U*^12^	*U*^13^	*U*^23^
Br1	0.01373 (9)	0.02465 (11)	0.01461 (10)	−0.00055 (7)	0.00604 (8)	0.00078 (7)
S1	0.0108 (2)	0.0186 (2)	0.0157 (2)	−0.00166 (16)	0.00767 (18)	−0.00202 (17)
O1	0.0163 (7)	0.0261 (8)	0.0243 (8)	0.0000 (5)	0.0145 (6)	−0.0017 (6)
O2	0.0145 (6)	0.0236 (7)	0.0181 (7)	−0.0042 (5)	0.0074 (6)	−0.0054 (6)
N1	0.0106 (7)	0.0202 (8)	0.0121 (7)	0.0001 (6)	0.0071 (6)	−0.0012 (6)
N2	0.0133 (7)	0.0189 (8)	0.0134 (7)	−0.0022 (6)	0.0098 (6)	−0.0043 (6)
C1	0.0136 (8)	0.0216 (9)	0.0150 (9)	−0.0010 (7)	0.0095 (7)	−0.0023 (7)
C2	0.0143 (8)	0.0140 (9)	0.0122 (8)	−0.0008 (6)	0.0085 (7)	−0.0008 (6)
C3	0.0147 (8)	0.0136 (8)	0.0133 (8)	0.0001 (6)	0.0097 (7)	0.0004 (7)
C4	0.0145 (8)	0.0118 (8)	0.0159 (9)	0.0000 (6)	0.0113 (7)	0.0002 (6)
C5	0.0180 (9)	0.0128 (8)	0.0132 (8)	−0.0001 (6)	0.0094 (7)	0.0003 (7)
C6	0.0152 (8)	0.0131 (8)	0.0126 (8)	0.0002 (6)	0.0065 (7)	0.0005 (6)
C7	0.0152 (8)	0.0165 (9)	0.0210 (9)	0.0013 (7)	0.0123 (8)	0.0004 (7)
C8	0.0167 (9)	0.0178 (9)	0.0181 (9)	−0.0007 (7)	0.0129 (8)	−0.0022 (7)
C9	0.0142 (8)	0.0121 (8)	0.0138 (8)	−0.0003 (6)	0.0092 (7)	0.0002 (6)
C10	0.0143 (8)	0.0138 (8)	0.0113 (8)	−0.0010 (6)	0.0088 (7)	−0.0008 (6)
C11	0.0129 (8)	0.0164 (9)	0.0123 (8)	−0.0004 (6)	0.0084 (7)	−0.0010 (7)
C12	0.0129 (8)	0.0179 (9)	0.0140 (9)	0.0008 (7)	0.0066 (7)	0.0003 (7)
C13	0.0288 (11)	0.0217 (10)	0.0161 (9)	−0.0002 (8)	0.0144 (9)	−0.0021 (8)
C14	0.0318 (12)	0.0256 (11)	0.0208 (10)	0.0016 (9)	0.0180 (10)	0.0017 (8)
C15	0.0152 (9)	0.0222 (10)	0.0218 (10)	0.0010 (7)	0.0103 (8)	0.0029 (8)
C16	0.0346 (12)	0.0185 (10)	0.0288 (11)	−0.0033 (8)	0.0220 (10)	−0.0048 (8)
C17	0.0315 (11)	0.0240 (11)	0.0242 (11)	−0.0025 (9)	0.0201 (10)	−0.0032 (8)
C18	0.0242 (11)	0.0233 (11)	0.0283 (12)	0.0004 (8)	0.0153 (10)	0.0046 (9)
C19	0.0105 (8)	0.0179 (9)	0.0106 (8)	0.0005 (6)	0.0060 (7)	−0.0007 (7)
C20	0.0238 (10)	0.0192 (10)	0.0204 (10)	0.0022 (7)	0.0164 (9)	0.0014 (8)
C21	0.0259 (10)	0.0152 (9)	0.0295 (11)	0.0014 (8)	0.0182 (10)	0.0009 (8)
C22	0.0249 (10)	0.0191 (10)	0.0266 (11)	0.0016 (8)	0.0159 (9)	−0.0063 (8)
C23	0.0271 (11)	0.0273 (11)	0.0236 (11)	−0.0020 (8)	0.0194 (9)	−0.0063 (8)
C24	0.0166 (9)	0.0203 (9)	0.0162 (9)	−0.0031 (7)	0.0111 (8)	−0.0020 (7)
C25	0.0152 (8)	0.0173 (9)	0.0169 (9)	−0.0033 (7)	0.0100 (8)	−0.0030 (7)
C26	0.0173 (9)	0.0142 (8)	0.0144 (9)	−0.0031 (7)	0.0101 (7)	−0.0045 (7)
C27	0.0233 (10)	0.0145 (9)	0.0168 (9)	−0.0010 (7)	0.0139 (8)	−0.0014 (7)
C28	0.0202 (9)	0.0174 (9)	0.0186 (9)	0.0019 (7)	0.0114 (8)	−0.0012 (7)
C29	0.0226 (10)	0.0196 (10)	0.0261 (11)	−0.0015 (8)	0.0176 (9)	−0.0043 (8)
C30	0.0289 (10)	0.0174 (9)	0.0199 (10)	−0.0032 (8)	0.0186 (9)	−0.0027 (7)
C31	0.0216 (9)	0.0176 (9)	0.0152 (9)	0.0004 (7)	0.0111 (8)	−0.0007 (7)

### Geometric parameters (Å, °)

**Table:**

Br1—C6	1.9002 (19)	C14—C15	1.385 (3)
S1—O2	1.4356 (15)	C14—H14	0.93
S1—O1	1.4364 (15)	C15—C16	1.392 (3)
S1—N1	1.6369 (16)	C15—C18	1.507 (3)
S1—C12	1.765 (2)	C16—C17	1.387 (3)
N1—C1	1.493 (2)	C16—H16	0.93
N1—C11	1.500 (2)	C17—H17	0.93
N2—C9	1.394 (2)	C18—H18A	0.96
N2—C10	1.451 (2)	C18—H18B	0.96
N2—H1N2	0.83 (3)	C18—H18C	0.96
C1—C2	1.520 (3)	C19—C24	1.391 (3)
C1—H1A	0.97	C19—C20	1.395 (3)
C1—H1B	0.97	C20—C21	1.388 (3)
C2—C10	1.519 (2)	C20—H20	0.93
C2—C3	1.527 (3)	C21—C22	1.385 (3)
C2—H2	0.98	C21—H21	0.93
C3—C19	1.519 (2)	C22—C23	1.383 (3)
C3—C4	1.533 (2)	C22—H22	0.93
C3—H3	0.98	C23—C24	1.393 (3)
C4—C5	1.397 (3)	C23—H23	0.93
C4—C9	1.411 (2)	C24—H24	0.93
C5—C6	1.388 (3)	C25—C26	1.516 (3)
C5—H5	0.93	C25—H25A	0.97
C6—C7	1.389 (3)	C25—H25B	0.97
C7—C8	1.380 (3)	C26—C27	1.396 (3)
C7—H7	0.93	C26—C31	1.398 (3)
C8—C9	1.405 (2)	C27—C28	1.397 (3)
C8—H8	0.93	C27—H27	0.93
C10—C11	1.529 (3)	C28—C29	1.388 (3)
C10—H10	0.98	C28—H28	0.93
C11—C25	1.537 (3)	C29—C30	1.389 (3)
C11—H11	0.98	C29—H29	0.93
C12—C17	1.387 (3)	C30—C31	1.391 (3)
C12—C13	1.392 (3)	C30—H30	0.93
C13—C14	1.388 (3)	C31—H31	0.93
C13—H13	0.93		
			
O2—S1—O1	120.33 (9)	C14—C13—C12	119.79 (19)
O2—S1—N1	106.05 (9)	C14—C13—H13	120.1
O1—S1—N1	106.73 (8)	C12—C13—H13	120.1
O2—S1—C12	106.91 (9)	C15—C14—C13	121.2 (2)
O1—S1—C12	108.03 (9)	C15—C14—H14	119.4
N1—S1—C12	108.31 (9)	C13—C14—H14	119.4
C1—N1—C11	110.57 (14)	C14—C15—C16	118.21 (19)
C1—N1—S1	118.47 (13)	C14—C15—C18	120.84 (19)
C11—N1—S1	117.12 (12)	C16—C15—C18	120.90 (19)
C9—N2—C10	113.13 (15)	C17—C16—C15	121.5 (2)
C9—N2—H1N2	116.8 (19)	C17—C16—H16	119.3
C10—N2—H1N2	114.8 (18)	C15—C16—H16	119.3
N1—C1—C2	103.47 (14)	C12—C17—C16	119.5 (2)
N1—C1—H1A	111.1	C12—C17—H17	120.3
C2—C1—H1A	111.1	C16—C17—H17	120.3
N1—C1—H1B	111.1	C15—C18—H18A	109.5
C2—C1—H1B	111.1	C15—C18—H18B	109.5
H1A—C1—H1B	109.0	H18A—C18—H18B	109.5
C10—C2—C1	101.27 (14)	C15—C18—H18C	109.5
C10—C2—C3	110.67 (15)	H18A—C18—H18C	109.5
C1—C2—C3	117.55 (15)	H18B—C18—H18C	109.5
C10—C2—H2	109.0	C24—C19—C20	118.40 (17)
C1—C2—H2	109.0	C24—C19—C3	120.53 (17)
C3—C2—H2	109.0	C20—C19—C3	121.07 (16)
C19—C3—C2	112.66 (15)	C21—C20—C19	120.90 (18)
C19—C3—C4	112.38 (15)	C21—C20—H20	119.6
C2—C3—C4	108.85 (15)	C19—C20—H20	119.6
C19—C3—H3	107.6	C22—C21—C20	119.98 (19)
C2—C3—H3	107.6	C22—C21—H21	120.0
C4—C3—H3	107.6	C20—C21—H21	120.0
C5—C4—C9	118.36 (17)	C23—C22—C21	119.88 (19)
C5—C4—C3	118.95 (16)	C23—C22—H22	120.1
C9—C4—C3	122.66 (16)	C21—C22—H22	120.1
C6—C5—C4	121.12 (17)	C22—C23—C24	120.05 (19)
C6—C5—H5	119.4	C22—C23—H23	120.0
C4—C5—H5	119.4	C24—C23—H23	120.0
C5—C6—C7	120.60 (18)	C19—C24—C23	120.78 (19)
C5—C6—Br1	119.63 (14)	C19—C24—H24	119.6
C7—C6—Br1	119.78 (14)	C23—C24—H24	119.6
C8—C7—C6	119.03 (17)	C26—C25—C11	108.30 (15)
C8—C7—H7	120.5	C26—C25—H25A	110.0
C6—C7—H7	120.5	C11—C25—H25A	110.0
C7—C8—C9	121.38 (17)	C26—C25—H25B	110.0
C7—C8—H8	119.3	C11—C25—H25B	110.0
C9—C8—H8	119.3	H25A—C25—H25B	108.4
N2—C9—C8	119.53 (16)	C27—C26—C31	118.40 (17)
N2—C9—C4	121.04 (16)	C27—C26—C25	121.08 (17)
C8—C9—C4	119.44 (17)	C31—C26—C25	119.92 (17)
N2—C10—C2	108.05 (15)	C26—C27—C28	120.74 (18)
N2—C10—C11	115.96 (15)	C26—C27—H27	119.6
C2—C10—C11	105.22 (14)	C28—C27—H27	119.6
N2—C10—H10	109.1	C29—C28—C27	120.12 (19)
C2—C10—H10	109.1	C29—C28—H28	119.9
C11—C10—H10	109.1	C27—C28—H28	119.9
N1—C11—C10	101.94 (13)	C28—C29—C30	119.60 (18)
N1—C11—C25	112.56 (15)	C28—C29—H29	120.2
C10—C11—C25	113.59 (15)	C30—C29—H29	120.2
N1—C11—H11	109.5	C29—C30—C31	120.21 (18)
C10—C11—H11	109.5	C29—C30—H30	119.9
C25—C11—H11	109.5	C31—C30—H30	119.9
C17—C12—C13	119.84 (19)	C30—C31—C26	120.83 (19)
C17—C12—S1	120.14 (15)	C30—C31—H31	119.6
C13—C12—S1	120.00 (15)	C26—C31—H31	119.6
			
O2—S1—N1—C1	170.23 (13)	C2—C10—C11—N1	30.32 (17)
O1—S1—N1—C1	40.79 (15)	N2—C10—C11—C25	−89.02 (19)
C12—S1—N1—C1	−75.31 (15)	C2—C10—C11—C25	151.67 (15)
O2—S1—N1—C11	−53.17 (15)	O2—S1—C12—C17	−159.73 (17)
O1—S1—N1—C11	177.39 (13)	O1—S1—C12—C17	−28.88 (19)
C12—S1—N1—C11	61.30 (15)	N1—S1—C12—C17	86.37 (18)
C11—N1—C1—C2	−18.81 (19)	O2—S1—C12—C13	18.59 (18)
S1—N1—C1—C2	120.42 (14)	O1—S1—C12—C13	149.44 (16)
N1—C1—C2—C10	36.53 (17)	N1—S1—C12—C13	−95.31 (17)
N1—C1—C2—C3	157.19 (15)	C17—C12—C13—C14	0.7 (3)
C10—C2—C3—C19	−166.61 (15)	S1—C12—C13—C14	−177.66 (16)
C1—C2—C3—C19	77.8 (2)	C12—C13—C14—C15	0.4 (3)
C10—C2—C3—C4	−41.27 (19)	C13—C14—C15—C16	−1.3 (3)
C1—C2—C3—C4	−156.90 (15)	C13—C14—C15—C18	176.2 (2)
C19—C3—C4—C5	−48.0 (2)	C14—C15—C16—C17	1.2 (3)
C2—C3—C4—C5	−173.54 (16)	C18—C15—C16—C17	−176.3 (2)
C19—C3—C4—C9	134.17 (17)	C13—C12—C17—C16	−0.7 (3)
C2—C3—C4—C9	8.7 (2)	S1—C12—C17—C16	177.61 (17)
C9—C4—C5—C6	−1.4 (3)	C15—C16—C17—C12	−0.2 (3)
C3—C4—C5—C6	−179.24 (16)	C2—C3—C19—C24	−113.59 (19)
C4—C5—C6—C7	−1.0 (3)	C4—C3—C19—C24	123.01 (18)
C4—C5—C6—Br1	179.26 (13)	C2—C3—C19—C20	66.4 (2)
C5—C6—C7—C8	1.7 (3)	C4—C3—C19—C20	−57.0 (2)
Br1—C6—C7—C8	−178.53 (14)	C24—C19—C20—C21	0.6 (3)
C6—C7—C8—C9	−0.1 (3)	C3—C19—C20—C21	−179.42 (18)
C10—N2—C9—C8	−155.20 (17)	C19—C20—C21—C22	0.0 (3)
C10—N2—C9—C4	25.2 (2)	C20—C21—C22—C23	−0.1 (3)
C7—C8—C9—N2	178.18 (18)	C21—C22—C23—C24	−0.5 (3)
C7—C8—C9—C4	−2.2 (3)	C20—C19—C24—C23	−1.1 (3)
C5—C4—C9—N2	−177.51 (17)	C3—C19—C24—C23	178.86 (18)
C3—C4—C9—N2	0.3 (3)	C22—C23—C24—C19	1.1 (3)
C5—C4—C9—C8	2.9 (3)	N1—C11—C25—C26	172.70 (15)
C3—C4—C9—C8	−179.28 (16)	C10—C11—C25—C26	57.5 (2)
C9—N2—C10—C2	−58.2 (2)	C11—C25—C26—C27	−99.7 (2)
C9—N2—C10—C11	−175.99 (15)	C11—C25—C26—C31	71.3 (2)
C1—C2—C10—N2	−166.58 (15)	C31—C26—C27—C28	−3.0 (3)
C3—C2—C10—N2	68.01 (18)	C25—C26—C27—C28	168.13 (18)
C1—C2—C10—C11	−42.12 (17)	C26—C27—C28—C29	0.5 (3)
C3—C2—C10—C11	−167.53 (14)	C27—C28—C29—C30	1.7 (3)
C1—N1—C11—C10	−6.89 (19)	C28—C29—C30—C31	−1.3 (3)
S1—N1—C11—C10	−146.72 (12)	C29—C30—C31—C26	−1.2 (3)
C1—N1—C11—C25	−128.95 (16)	C27—C26—C31—C30	3.3 (3)
S1—N1—C11—C25	91.22 (17)	C25—C26—C31—C30	−167.86 (18)
N2—C10—C11—N1	149.63 (15)		

### Hydrogen-bond geometry (Å, °)

**Table:**

*D*—H···*A*	*D*—H	H···*A*	*D*···*A*	*D*—H···*A*
C1—H1A···O1	0.97	2.53	2.917 (3)	104
C13—H13···O2	0.93	2.56	2.914 (3)	103
C28—H28···O2^i^	0.93	2.57	3.207 (3)	126
N2—H1N2···Cg3	0.83 (3)	2.53 (3)	3.289 (2)	152 (3)
C3—H3···Cg3^ii^	0.98	2.98	3.924 (2)	162
C18—H18A···Cg2^iii^	0.96	2.94	3.737 (3)	141
C21—H21···Cg1^iv^	0.93	2.80	3.676 (2)	158

Symmetry codes: (i) *x*−1, −*y*+1/2, *z*−1/2; (ii) *x*, −*y*+1/2, *z*−1/2; (iii) −*x*+2, −*y*, −*z*+2; (iv) −*x*+1, −*y*, −*z*+1.
